# Improving Self-Care Management in Patients with Breast Cancer through Health Literacy Promotion

**DOI:** 10.4314/ejhs.v31i1.10

**Published:** 2021-01

**Authors:** Isa Ahmadzadeh, Mohammadhiwa Abdekhoda, Mahmud Bejani

**Affiliations:** 1 School of Health Management and Medical Informatics, Tabriz University of Medical Sciences, Tabriz, Iran; 2 Department of Medical Library and Information Sciences, Iranian Center of Excellence in Health Management (IceHM), School of Health Management and Medical Informatics, Tabriz University of Medical sciences, Tabriz, Iran; 3 Hematology and Oncology Research Center, Tabriz University of medical sciences. Tabriz, Iran; 4 Student Research Committee of Tabriz University of Medical Sciences, Faculty of Management and Information, Tabriz University of Medical Sciences, Tabriz, Iran

**Keywords:** Health Literacy, Self-care, Breast Cancer, Breast Neoplasms, Self-care Maintenance, Self-care Management, Self-care Confidence

## Abstract

**Background:**

Self-care is one of the most important principles of life and the successful treatment of patients diagnosed with cancer. Also, the first step and the most effective factor for self-care is health literacy. Thus, the aim of this study is to evaluate the relationship between level of health literacy and self-care ability in patients diagnosed with breast cancer.

**Method:**

Using an analytical-descriptive approach, the present study was conducted using a random access method among 120 patients diagnosed with breast cancer referring to Imam Reza Hospital of Tabriz University of Medical Sciences, during 2019. The data was gathered using two standard self-care questionnaires in patients diagnosed with breast cancer (SCHFIV6.2) and Health Literacy for Iranian Adults (HELIA). Data analysis was conducted using SPSS _v22_, through Pearson correlation tests and linear regression analysis.

**Results:**

The results indicate a positive and significant relationship among dimensions of health literacy including access, reading, appraisal, decision, and understanding, and dimensions of self-care including self-care maintenance, self-care management, and self-care confidence. Other findings show that reading, access and decisions have a direct and significant effect on self-care ability (P-value ≤0.01).

**Conclusion:**

With regard to the direct and significant relationship of health literacy and self-care dimensions in patients diagnosed with breast cancer, enhancing their health literacy can result in improved self-care among them. In addition, due to the increasingly high prevalence of this disease among women, improving their health literacy can be effective in the treatment of this disease or in enhancing their quality of life.

## Introduction

On the verge of the 21^st^ century, the most prominent occurrence that health service providers and societies are faced with is the increasing prevalence of chronic diseases. This is while individuals' health habits and behaviors can be extremely effective in the severity and diagnosis rate of these diseases. Self-care is one of the most important principles of life and is considered a successful factor in treating patients diagnosed with chronic diseases such as breast cancer. This is while one of the most effective factors in self-care is health literacy (1,2).

Breast cancer is one of the important diseases with a high prevalence rate among women (98%) with the second highest mortality rate after lung cancer. With advancements in science and technology, today the screening and detection of breast cancer can be easily done at the onset of the disease. After diagnosis, patients' behavior and type of lifestyle has a direct effect on increased life expectancy among them (3). Health literacy includes the ability to identify the need for health information, appropriate information sources and the means to use them for retrieving related information. It is also necessary to assess the quality of information and its applicability in a certain situation, and to analyze, understand and apply this information for appropriate decisionmaking in health-related issues. Health literacy includes cognitive and social skills that determine individuals' ability and motivation in accessing, understanding and applying information in order to maintain and promote their health (4).

Breast cancer is defined as changes in the uncontrollable growth of cells in the breast tissue, and this abnormal growth occurs in the mammary glands (lobules) or ducts that join the lobules to the nipples. The prevalence of breast cancer in the United States and Europe is double that of Asian countries and is on the rise all across the globe; even though its prevalence in Asia is less than that of western countries. Based on the latest statistics of the Center for Cancer Research in Iran, approximately 8500 new cases of breast cancer are reported annually nationwide, and 1400 lose their lives as a result of this disease. Also, about 40000 individuals are currently living with this disease in the country. Thus, considering the high prevalence of breast cancer and the infection and mortality rate across the world, it seems that the best method to control it is prevention. Primary prevention is possible by abstaining from the hazardous factors identified. Secondary prevention should be implemented by means of different screening methods for initial identification of the tumor and its timely treatment. This can have an effective role in reducing the socio-economic harm resulting from cancer in the family and in society at large.

Considering the significance of self-care in breast cancer patients and with regard to the self-management nature of this disease, over 90% of the disease is managed by the patient (5). For success in the treatment process, the present research was conducted with the aim of evaluating the relationship between health literacy and different aspects of self-care in breast cancer patients. Hence, the relationship between health literacy and different dimensions of self-care including self-care maintenance, self-care management, and self-care confidence were identified.

## Methods

The current research was a descriptive, correlational study carried out in a specified duration of time and with practical goals. The statistical population included patients diagnosed with breast cancer of grades 2 and 3, as confirmed by a specialist, and all having reported to Imam Reza Hospital in Tabriz for treatment from August to December 2019. Sampling was conducted using a random access method. The data collection instrument included two valid questionnaires: the Self-care behaviors in patients diagnosed with breast cancer and the Health Literacy for Iranian Adults questionnaires.

The self-care questionnaire for clinical assessment of self-care for patients diagnosed with breast cancer uses the Self-Care Heart Failure Index v6.2 (SCHFI), which was obtained by the self-care index questionnaire designed by Riegel et al. in 2009 (6). The SCHFI includes 21 items in three subscales of self-care maintenance (9 items), self-care management (5 items), and self-care self-confidence (7 items), with responses based on the 5-point Likert scale from completely agree to completely disagree (from 1 to 5, respectively). This questionnaire is converted to a score of 100 in each subscale, in which higher scores indicate better self-care, and scores higher than 70 indicate adequate self-care. The validity and reliability of this questionnaire were determined in different studies, in which the validity of this questionnaire was 83%. In order to determine the reliability of the questionnaire, internal correlation was used for which the Cronbach alpha was obtained at greater than 80. In the study by Reigel et al. in 2009, after updating their questionnaire, they reported the reliability to be higher than 70 (14).

In order to collect data related to health literacy, the Health Literacy for Iranian Adults (HELIA) questionnaire was used. The questionnaire used included 33 items in which the health literacy level was evaluated in five dimensions of Reading (5 items), Access (6 items), understanding (7 items), appraisal (4 items), and decision (12 items). The responses were also based on the five point Likert scale, specified from “always” to “never”. Calculating the score for this instrument was based on “single item score” and “total score”, whereas the score for this instrument (single item score and total score) is obtained from the sum score of all responses. Structural validity of the instrument using exploratory factor analysis shows that the questionnaire with 33 items in five dimensions had adequate validity, explaining an overall 53.2% of changes observed. In addition, the reliability was approved by obtaining Crobach's alpha (83 90), which is an acceptable reliability (15).

Finally, after data collection, SPSS _v22_ was used to analyze the data by calculating descriptive statistics indices (frequency, average variance, minimum and maximum standard deviation), inferential statistics, and Pearson correlation and linear regression tests.

## Results

Overall, 120 patients diagnosed with breast cancer with an average age range of 43.5±5.3 were recruited for the study. Considering the type of disease that is dependent on gender, all participants were females. Approximately 11.7% of the women were single, and 88.3% were married. Most participants had elementary school education (68%), 18% were illiterate, 14% had middle school education, and 17% had diplomas. About 25 percent of the participants mainly obtained their information by asking questions from physicians and healthcare workers, and 25% did not know where they had obtained their information. Patients obtained the necessary information from the healthcare staff, by asking friends and acquaintances, through television and radio, booklets and educational brochures, and the Internet. None of the participants mentioned getting health information through Interactive Voice Response calls or satellite networks.

Table 1 shows the correlation between health literacy and self-care dimensions. The findings in this table indicated a positive, meaningful and strong correlation among health literacy dimensions, including access, reading, decision, appraisal, understanding, and self-care dimensions, including self-care maintenance, self-care management, and self-care confidence. That is, with an increase in health literacy, self-care ability in patients increases.

**Table 1:** Correlation between Health literacy dimensions and self-care dimensions

Health literacy Dimensions	Reading	Access	Understanding	Appraisal	Decision
Self- care dimensions					
Self-care maintenance	0.74*	0.83*	0.78*	0.66*	0.79*
Self-care management	0.66*	0.78*	.75*	0.56*	0.77*
Self-care confidence	0.63*	0.77*	0.74*	0.59*	0.75*

* (p-value<0.01)

Table 2 shows the regression coefficient between health literacy and self-care dimensions. The results indicated that reading, access and decision have a direct and significant effect on self-care maintenance. Other findings in this table show that access and decision have a direct and significant effect on self-care management and self-care confidence.

**Table 2:** The regression coefficient between health literacy dimensions and self-care dimensions

Health literacy Dimensions		Reading	Access	Understanding	Appraisal	Decision
Self-care dimensions						
Self-care maintenance	β	0.18	0.38	0.07	0.12	0.32
	p-value	0.03	0.01	0.56	0.06	0.01
Self-care management	β	0.05	0.39	0.05	0.01	0.34
	p-value	0.57	0.01	0.72	0.91	0.01
Self-care confidence	β	0.03	0.44	0.05	0.23	0.28
	p-value	0.79	0.01	0.07	0.09	0.02

Table 3 shows the prediction rate for self-care dimensions using health literacy dimensions. In addition, the findings in this table indicate that reading controls about 59% of self-care maintenance variance, access controls about 14%, and decision controls 10% of this variance. Other findings show that decision controls 41% of the self-care management variance.

**Table 3:** Coefficient of determination analysis

Self-care Health dimensions	Self-care maintenance			Self-care management			Self-care confidence		
Literacy Dimensions									
	R	R^2^	sig	R	R^2^	sig	R	R^2^	sig
Reading	0.74	0.59	0.03	0.66	0.55	0.57	0.63	0.76	0.75
Access	0.83	0.14	0.00	0.78	0.05	0.01	0.77	0.02	0.00
Understanding	0.78	0.35	0.56	0.75	0.42	0.72	0.74	0.41	0.70
Appraisal	0.66	0.06	0.56	0.56	0.00	0.91	0.59	0.05	0.23
Decision	0.79	0.10	0.00	0.77	0.41	0.00	0.75	0.08	0.23

The results in this table indicate the positive and significant relationship between health literacy dimensions and self-care maintenance. Thus, the greatest relationship was calculated between the access dimension and self-care dimension at 0.83%, followed by the decision dimension at 0.79%, understanding at 0.78%, reading at 0.74% and finally appraisal at 0.66%.

## Discussion

If there is a significant relationship between health literacy and self-care ability, it can be concluded that in order to increase self-care ability in patients diagnosed with breast cancer, health literacy among these patients must be enhanced with the help of arrangements made by policymakers in the field of health. The current study was carried out in order to approve or reject this hypothesis and evaluate the relationship between health literacy and self-care ability in patients diagnosed with breast cancer.

The results of this study show that there is a direct and significant relationship between five dimensions of health literacy including reading, access, understanding, appraisal, and decision; and self-care dimensions including self-care maintenance, self-care management, and self-care confidence in patients diagnosed with breast cancer. That is, patients with a higher level of health literacy show better self-care behavior and have better performance regarding self-care maintenance, self-care management, and self-care confidence.

In the following details, in order to further discuss the issue and review previous studies in this field, the results of our study will be compared with those studies and finally necessary conclusions will be made in order to approve or reject our hypotheses.

In the study conducted by Dennison et al. in 2011 on the relationship between health literacy and having adequate knowledge regarding heart failure and self-care confidence in 95 heart failure patients, it was shown that 42% of patients taking part in the study did not have adequate health literacy, 39% had adequate health literacy, and 16% had marginal health literacy. The average score obtained for this group in the Self-care Heart Failure Index (SCHFI) was 56.82±17.12 for self-care maintenance, 63.64±18.29 for self-care management, and 65.02±02 for self-care confidence. Among the three above-mentioned groups (adequate, inadequate and marginal health literacy) those with adequate health literacy had good knowledge of heart failure compared to those with inadequate or marginal health literacy. Moreover, self-care maintenance and self-care management were not affected by health literacy rate. Patients with high health literacy rate had good knowledge related to heart failure and also high self-confidence (7). The results and findings of this research were consistent with the study by Dennison et al., specifically with regard to self-care confidence and health literacy.

In the study by Chen et al. (2011) (17), the relationship between health literacy and self-care among 49 patients with heart failure was evaluated, indicating that self-care management had a direct relationship with health literacy rate, which is similar to our findings. However, their results showed that there is no relationship between health literacy and self-care confidence, while in this study, we came to the conclusion that this relationship is positive and significant. Chen et al. also found that there is an inverse relationship between health literacy and self-care maintenance (p=0.001, -0.573), while we came to the conclusion that the relationship between these two variables is positive and significant (8).

In the study by Mohammadpour et al. (2018), 354 patients diagnosed with hypertension were evaluated. It was found that there was a significant correlation between overall score for health literacy and self-care behavior in following a nutritional diet (r=0.092, p=0.085), following a pharmaceutical diet (r=0.007, p=0.038) and physical activity as another aspect of self-care (r=0.122, p=0.022) (9). The results of this study also confirmed the correlation between health information literacy dimensions and self-care behavior in breast cancer patients.

In the study by Najimi et al. (2020) entitled “Health literacy and self-care in reproductive age”, health literacy was divided into three groups of inadequate (28%), marginal (23%) and adequate (49%). Results of the variance analysis test showed that the score for self-care, body care (F=9.35, p<0.001) and stress management (F=5.38, p=0.007) were significant at various levels of health literacy (adequate, marginal, inadequate). The results of this research indicated that patients with inadequate health literacy levels compared to those with adequate levels of health literacy had a lower average score for self-care in body care and stress management (10). The results of the current study on health literacy and self-care are in line with this research.

In the study by Alizadeh Aghdam et al. (2017) conducted on 414 participants above 15 years of age in Tabriz, the relationship between physical health dimensions, mental health, and social health were evaluated using the standard General Health Questionnaire (GHQ) with health literacy dimensions including reading, access, understanding, appraisal, and decision-making. The findings indicate the positive and significant relationship between level of education and self-care (p=0.019). Also, a positive and significant relationship was obtained between health literacy variables and mental health (p=0.000), and self-care and mental health (p=0.000). The results of this study indicate a positive and significant relationship between self-care and mental health in patients, while with an increase in the level of self-care, mental health also increases (11). The results of our study were similar to the results of the study by Alizadeh Aghdam et al.

The study by Wolf et al. (2014) on 283 outpatients referring to a hospital in the United States indicated that patients with lower health literacy showed weaker physical performance and inadequate mental health compared to their counterparts, while the prevalence of chronic diseases, lack of daily activities and weak physical performance was greater in patients with inadequate health literacy, and these patients had inadequate mental health compared to other patients (12).

In the study by Schillinger et al. (2002) which was conducted with the participation of 408 diabetic patients referring to two clinics in San Francisco, using the Test of Functional Health Literacy in Adults (s-TOFHLA), it was found that patients with inadequate health literacy had fewer skills and abilities in diabetic self-care, and the risk of retinopathy was higher among these patients (13). In our study, we also found that breast cancer patients with adequate health literacy were more successful in self-care maintenance and management.

Overall, it can be concluded that the findings of the current study are in line with most previous research indicating the positive and significant relationship between the three dimensions of self-care and five dimensions of health literacy. That is, if health literacy among patients diagnosed with breast cancer is enhanced, self-care will increase in three dimensions of self-care maintenance, self-care management, and self-care confidence. Therefore, health literacy and its enhancement is one of the fundamental strategies in enhancing self-care abilities in breast cancer patients, hence improving the quality of life in these patients, facilitating the management and leadership of this disease for health care personnel, while also reducing costs to a great extent.
